# Association of NAP1L1 polymorphism with clinicopathological progression in oral squamous cell carcinoma

**DOI:** 10.7150/jca.132751

**Published:** 2026-06-17

**Authors:** Ming-Ju Hsieh, Hsin-Yu Ho, Yu-Sheng Lo, Min-Yun Kao, Chia-Chieh Lin, Yi-Ching Chuang, Mu-Kuan Chen

**Affiliations:** 1 Oral Cancer Research Center, Changhua Christian Hospital, Changhua 50006, Taiwan.; 2 Graduate Institute of Clinical Medicine, College of Medicine, National Chung Hsing University, Taichung 402202, Taiwan.; 3 Graduate Institute of Biomedical Sciences, China Medical University, Taichung 404328, Taiwan.; 4 Department of Dental Technology and Materials Science, Central Taiwan University of Science and Technology, Taichung 406, Taiwan.; 5 Department of Otorhinolaryngology, Head and Neck Surgery, Changhua Christian Hospital, Changhua 50006, Taiwan.

**Keywords:** NAP1L1, polymorphism, oral cancer, lymph node, betel, cigarette smoking

## Abstract

**Objective:**

Nucleosome assembly protein 1-like 1 (NAP1L1) plays an important role in the development and progression of several types of cancer.

**Subject and Methods:**

This study investigated the association between NAP1L1 polymorphisms, particularly rs11180813, and susceptibility to oral squamous cell carcinoma (OSCC).

**Results:**

Our study showed that betel quid chewing and cigarette smoking were significantly associated with OSCC risk (p < 0.001). No significant correlation was found between NAP1L1 SNPs and overall OSCC susceptibility. However, among OSCC patients, patients with rs11180813 variant genotypes (GC/CC) were associated with lymph node metastasis (adjusted OR = 1.565, 95% CI = 1.042-2.350, p = 0.031) and poor tumor differentiation (adjusted OR = 2.284, 95% CI = 1.126-4.635, p = 0.022). In buccal OSCC, the rs11180813 variant was associated with lymph node metastasis (adjusted OR = 2.349, 95% CI = 1.213-4.549, p = 0.011). TCGA analysis revealed that high NAP1L1 expression correlated significantly with lymph node metastasis and poor tumor differentiation (p < 0.0001).

**Conclusion:**

These findings suggest that NAP1L1 and its rs11180813 polymorphism may be associated with certain clinicopathological characteristics of OSCC, particularly lymph node metastasis and tumor differentiation. Further studies are required to validate their clinical significance.

## 1. Introduction

Oral Squamous Cell Carcinoma (OSCC) incidence is continually increasing and is expected to rise by 30 % by 2030 1. It is currently the fourth most common cancer affecting men in Taiwan 2. Despite advances in treatment modalities, the 5-year survival rate of oral cancer was 50%-60% 3, 4. Alcohol consumption, betel nut chewing, smoking, human papillomavirus (HPV) infection, and environmental factors are the most recognized etiological factors for OSCC occurrence 5, 6. Early detection of primary tumors and timely treatment are key determinants of patient survival 7.

Human Nucleosome assembly protein 1-like (NAP1L) family contains NAP1L1- NAP1L5 8. Among all members of the NAP1L family, NAP1L1 has a higher nucleosome assembly capability 9. NAPs are members of a highly conserved histone chaperone family found in animals, plants, and yeast 10, 11. NAP1L1 is ubiquitously expressed in human tissues and cell lines, whereas increased levels were frequently found in high proliferating cells 12. NAP1L1 is a potential tumor oncogene expressed in endometrial cancer, breast cancer, pancreatic neuroendocrine neoplasm and colon cancer 10, 13-15. NAP1L1 induces HCC cell proliferation by inducing PI3K/AKT/mTOR pathway 8. In nasopharyngeal carcinoma (NPC), NAP1L1 exerts oncogenic effects by activating HDGF/c-JUN/CCND1 signaling pathway 13. Collectively, these studies indicate that NAP1L1 plays an important role in tumor development. However, the mechanisms driving OSCC development and the role of NAP1L1 SNPs remains poorly understood. Therefore, in this study we investigated the relationship between NAP1L1 genetic polymorphisms and OSCC risk and clinical outcomes in Taiwanese patients.

## 2. Materials and Methods

### 2.1 Patients and specimens

The samples for this study were collected at Changhua Christian Hospital. Ethical approval for this study was obtained from the Institutional Review Board (IRB) at Changhua Christian Hospital (CCH) under the number 130616. The research group included 910 patients diagnosed with OSCC and 672 cancer-free patients in the control group in Changhua Christian Hospital from 2014 to 2023. A total of 1582 cases were collected for this study, and all patients participating in the study signed a written informed consent form before the start of the project. There was no geographical difference between the study populations, and they all lived in Han Chinese communities. We obtained statistical data on age and personal habits (including betel nut chewing, smoking and alcohol consumption) from medical documents. Additionally, AJCC No. 8 was also used to discuss the judgment of the clinical stage, tumor/lymph node/metastasis (TNM) stage and degree of cell differentiation. For NAP1L1 polymorphisms, venous blood samples were collected by the investigator and stored in tubes containing K3-Ethylene diamine tetra acetic acid (EDTA). The blood samples were then cryogenically centrifuged and stored in a -80 °C laboratory freezer for analysis.

### 2.2. Functional NAP1L1 SNP selection

Due to the few references to NAP1L1 SNPs, we selected other NAP1L1 SNPs in Asian populations by using the ABI probe database and filtered them using linkage disequilibrium (LD) and minor allele frequency (MAF). The NAP1L1 SNPs were selected using an ABI SNP browser, and we then excluded LD sites through the LD-LINK website. Subsequently, SNPs with a minor allele frequency lower than 5% were excluded based on the NIH Variation Viewer. This eliminates options with LD-LINK scores higher than 0.8 and MAF percentages lower than 5%. After eliminating the above conditions, three NAP1L1 polymorphism sites were selected, namely rs11180813, rs75354731 and rs76535500, and the MAF of each polymorphism were 6.5%, 9.1% and 10.1%, respectively. The three NAP1L1 SNPs, rs11180813 (G/C), rs75354731 (G/A), and rs76535500 (T/G), were included in the analysis model.

### 2.3. DNA extraction and genotyping of NAP1L1 SNPs by real-time PCR

Similar to our previous research, we used DNA extraction, preservation, and analysis techniques. Whole blood samples were collected from patients into sterile tubes containing EDTA, which were immediately centrifuged and stored at -80 °C. The genomic DNA was extracted from peripheral blood leukocytes using a QIAamp DNA blood mini kit, and then dissolved in TE buffer and stored at -20 °C. Quantification was based on measuring the optical density at a wavelength of 260 nm. The three NAP1L1 polymorphisms rs11180813 (G/C), rs75354731 (G/A) and rs76535500 (T/G) were determined by real-time quantitative PCR using the ABI StepOne real-time PCR system (Applied Biosystems, Foster City, CA, USA) and were analyzed using StepOne Software v2.3. Each reaction mixture contained 2.5 µL of TaqMan Genotyping Master Mix, 0.125 µL TaqMan probe mix, and 30 ng genomic DNA was used to create each reaction, resulting in a final volume of 5 µL. Real-time PCR was conducted with an initial denaturation step at 95 °C for 10 min, followed by 40 amplification cycles at 95 °C for 15 s and 60 °C for 1 min.

### 2.4. Bioinformatics analysis of NAP1L1 expression

To further investigate the clinical relevance of NAP1L1 expression, we analyzed transcriptomic and clinical data from The Cancer Genome Atlas (TCGA) Head and Neck Squamous Cell Carcinoma (HNSCC) cohort using the UCSC Xena functional genomics browser. NAP1L1 mRNA expression levels were compared among different clinical subgroups according to tumor stage, tumor size (T status), lymph node status (N status), and tumor differentiation. Statistical comparisons were performed using the Mann-Whitney U test. It should be noted that the TCGA-HNSCC dataset includes tumors arising from multiple anatomical sites of the head and neck region, including the oral cavity, oropharynx, hypopharynx, and larynx.

### 2.5. Statistical analysis

We used IBM SPSS Statistics v22.0 (IBM, Armonk, NY, USA) to conduct the analyses in our study, similarly to previous papers. First, the demographic and laboratory data between the non-OSCC group and the OSCC group were shown using descriptive analysis including mean, standard deviation (SD) and percentage, and evaluated using a Mann-Whitney U test to test the difference between the two groups. We were able to estimate the adjusted odds ratios (AORs) using a multiple logistic regression model in SPSS statistical analysis after controlling for betel nut chewing, alcohol consumption, and tobacco use to exclude possible influencing environmental factors. Logistic regression models were then used to analyze the AORs and associated 95% CI of the NAP1L1 SNP polymorphism distribution between non-OSCC and OSCC populations. We further categorized patients with oral squamous cell carcinoma (OSCC) into different locations and analyzed the correlation between NAP1L1 SNP rs11180813 and clinicopathological features of OSCC to generate adjusted odds ratios (AORs) with 95% confidence intervals. NAP1L1 level variation in TCGA's HNSCC data set was compared with the Mann-Whitney U test. A p-value below 0.05 was defined as of statistical significance. Because this study was designed as an exploratory candidate SNP association study, multiple comparison correction methods were not initially applied, and the findings should therefore be interpreted with caution.

## 3. Results

### 3.1 Demographic and clinical characteristics

The clinical characteristics of the OSCC patients are shown in Table 1. A total of 910 OSCC patients and 672 cancer-free controls were involved in this study. About 50% of participants in each group were aged below 55 years. Our results indicate that betel nut chewing and cigarette smoking are significantly associated with an increased risk of OSCC (p < 0.001). No significant differences were observed between the control and OSCC groups with respect to age or alcohol consumption. The clinicopathological characteristics of OSCC patients, including clinical stage, TNM status, and cell differentiation, are summarized in Table 1. The high expression of NAP1L1 was significantly associated with lymph node metastasis (P < 0.0001) and poor tumor differentiation (P < 0.0001). Interestingly, the TCGA expression analysis demonstrated trends consistent with the SNP association results, particularly regarding lymph node metastasis and tumor differentiation, suggesting a potential relationship between NAP1L1 dysregulation and aggressive clinicopathological features in OSCC.

### 3.2 Association between NAP1L1 polymorphisms and OSCC risk

To analyze the genotype distribution and association between OSCC and NAP1L1 SNPs, three SNPs of the NAP1L1 gene (rs11180813, rs75354731, and rs76535500) were evaluated in the control group and OSCC group (Table 2). For the rs11180813 polymorphism, the GG genotype was the most prevalent in both controls (88.1%) and OSCC patients (87.0%) and was therefore used as the reference genotype. Neither the heterozygous GC genotype nor the homozygous CC genotype was significantly associated with OSCC risk (GC: Odd ratio (OR) = 1.087, 95% CI = 0.798-1.481, p = 0.596; CC: OR = 1.495, 95% CI = 0.372-6.002, p = 0.571). Similarly, analysis of the combined GC + CC genotypes did not reveal a significant association with OSCC susceptibility in either odds ratio or adjusted odds ratio models after controlling for betel nut chewing, alcohol consumption, and tobacco use. For rs75354731, the GG genotype was also the predominant genotype in both the control (83.8%) and OSCC (81.0%) groups. Compared with the GG genotype, neither the GA nor the AA genotype showed a statistically significant association with OSCC risk (GA: OR = 1.183, 95% CI = 0.903-1.549, p = 0.223; AA: OR = 1.833, 95% CI = 0.642-5.234, p = 0.257). Consistent results were observed when GA and AA genotypes were combined, and the associations remained non-significant after adjustment for potential confounders. Similarly, analysis of rs76535500 demonstrated no significant differences in genotype distributions between controls and OSCC patients. The TT genotype served as the reference, and neither the TG nor the GG genotype was associated with OSCC risk in crude or adjusted analyses. The combined TG + GG genotype also failed to show a statistically significant association with OSCC susceptibility.

### 3.3 NAP1L1 polymorphism and clinicopathological characteristics in patients with OSCC

The correlation between genotype and clinicopathological characteristics in OSCC patients was further investigated. Table 3 summarizes the clinical correlations of rs11180813 genotypes in OSCC patients. Although rs11180813 was not associated with tumor stage, tumor size, or distant metastasis, patients harboring the GC + CC genotypes exhibited a significantly higher risk of lymph node metastasis (OR = 1.546, 95% CI: 1.032-2.316; p=0.035; AOR = 1.565, 95% CI: 1.042-2.350; p = 0.031). Additionally, these variant genotypes were more frequently detected in moderately or poorly differentiated tumors (OR = 2.291, 95% CI: 1.131-4.642; p=0.021; AOR = 2.284, 95% CI: 1.126-4.635; p = 0.022). It should be noted that the frequency of the rs11180813 variant genotype was relatively low in this cohort. Nevertheless, a statistically significant association was observed between the variant genotype and certain clinicopathological characteristics, including lymph node metastasis and tumor differentiation.

### 3.4 NAP1L1 rs11180813 and clinical features in buccal OSCC

The relationships between the NAP1L1 rs11180813 polymorphism and clinicopathological characteristics in patients with buccal OSCC are summarized in Table 4. The prevalence of lymph node involvement was markedly higher among patients with GC + CC genotypes (40.9%) than among those with the GG genotype (22.7%). Logistic regression analysis demonstrated that GC + CC genotypes showed two-fold increased risk of lymph node metastasis (OR = 2.356, 95% CI = 1.218-4.557, p = 0.011), and this association remained statistically significant after adjustment for betel nut chewing, alcohol consumption, and tobacco use (AOR = 2.349, 95% CI = 1.213-4.549, p = 0.011). No significant associations were found with clinical stage, tumor size, distant metastasis, or cell differentiation. This suggests a potential site-specific role of rs11180813 in promoting nodal spread in buccal OSCC.

## 4. Discussion

Previous studies have demonstrated that OSCC progression is a multistep process influenced by both genetic susceptibility and environmental exposures 16. Recent evidence indicates that NAP1L1 is involved in the pathogenesis of several cancer types, including head and neck squamous cell carcinoma (HNSCC) 17, 18. Liu et al. (2021) reported that NAP1L1 mRNA expression is significantly upregulated in HNSCC tissues 16. In the present study, we investigated the association between NAP1L1 genetic polymorphisms and OSCC susceptibility and clinicopathological characteristics in a Taiwanese population. Although NAP1L1 polymorphisms were not associated with overall OSCC risk, our findings demonstrate that the rs11180813 variant genotype is significantly associated with aggressive clinicopathological features, particularly lymph node metastasis and poor tumor differentiation.

NAP1L1 is a histone chaperone involved in nucleosome assembly and chromatin remodeling, processes that regulate gene transcription and influence cellular proliferation, invasion, and differentiation. Previous studies have reported that NAP1L1 promotes tumor progression in several malignancies through transcriptional regulation and activation of oncogenic signaling pathways, including PI3K/AKT/mTOR 13, 16. However, most previous studies have focused on the functional role or expression level of NAP1L1 in tumor development. In contrast, the present study examined whether genetic variations in the NAP1L1 gene contribute to OSCC susceptibility and tumor progression. These findings suggest that NAP1L1 may influence tumor progression by regulating transcriptional programs associated with cancer cell proliferation, invasion, and differentiation.

Consistent with previous epidemiological studies 6, our data confirm that betel nut chewing and cigarette smoking are major risk factors for OSCC development (Table 1). OSCC is widely recognized as the result of complex interactions between genetic susceptibility and environmental exposures. In Taiwan, betel quid chewing, cigarette smoking, and alcohol consumption are well-established risk factors for OSCC. Although these environmental factors were adjusted for in the regression analyses to minimize potential confounding effects, genetic susceptibility loci may still interact with environmental carcinogens to influence tumor progression. Chronic exposure to betel quid-related carcinogens has been reported to induce genomic instability and epigenetic alterations in oral epithelial cells, which may cooperate with genetic variants to promote malignant transformation and tumor aggressiveness.

Interestingly, the rs11180813 variant genotype was associated with lymph node metastasis and tumor differentiation among OSCC patients. Notably, the clinicopathological associations observed in the SNP analysis were partially consistent with the TCGA expression analysis, in which elevated NAP1L1 expression was also associated with lymph node metastasis and poor tumor differentiation. Although the TCGA cohort was not restricted exclusively to OSCC cases, these findings collectively support a potential role of NAP1L1 in tumor progression and aggressive tumor behavior. Previous studies have suggested that chromatin remodeling proteins may contribute to epithelial mesenchymal transition, metastatic dissemination, and tumor microenvironment regulation in HNSCC 16. Given the role of NAP1L1 in nucleosome assembly and transcriptional regulation, it is possible that altered NAP1L1 activity may indirectly influence metastatic-associated gene expression programs. Nevertheless, the precise biological mechanisms remain unclear and require further functional investigation. This polymorphism is located within an intronic region of the NAP1L1 gene. Although intronic variants do not directly alter protein sequences, increasing evidence suggests that they may influence gene expression through regulatory mechanisms such as transcription factor binding, RNA splicing, or chromatin accessibility. Given the role of NAP1L1 in chromatin remodeling and transcriptional regulation, it is possible that the rs11180813 variant may affect NAP1L1 expression or activity, thereby contributing to tumor progression and metastatic potential in OSCC. Interestingly, no significant association was observed between the rs11180813 polymorphism and overall tumor stage. One possible explanation is that tumor stage represents a composite clinical classification that incorporates multiple factors, including tumor size, lymph node involvement, and distant metastasis. Because the rs11180813 variant was primarily associated with lymph node metastasis rather than tumor size or distant metastasis, its effect may not be reflected in the overall tumor stage classification.

Notably, the association was more pronounced in patients with buccal OSCC, where the rs11180813 variant genotype conferred approximately a two-fold increased risk of lymph node metastasis. This site-specific effect may reflect distinct etiological mechanisms and tumor microenvironments in buccal mucosa, which is frequently exposed to betel nut-related carcinogens 19. These findings suggest that the biological impact of NAP1L1 polymorphisms may vary depending on tumor location and environmental exposure.

Lymph node metastasis is a critical prognostic factor in OSCC 20. Genetic markers associated with nodal involvement may provide additional information for understanding OSCC progression. In this study, patients carrying the rs11180813 variant genotypes (GC/CC) exhibited a higher likelihood of lymph node metastasis and poorer tumor differentiation, suggesting that this polymorphism may help identify patients with more aggressive tumor behavior. Such genetic information may complement conventional clinicopathological factors to improve early identification of high-risk OSCC patients and guide individualized management strategies.

Several limitations of this study should be acknowledged. First, this study was conducted in a Taiwanese population consisting predominantly of Han Chinese individuals, and therefore the findings may not be directly generalizable to other ethnic groups. In addition, the TCGA dataset used in this study consisted of heterogeneous HNSCC subtypes rather than exclusively oral cavity tumors, which may limit the specificity of the bioinformatic findings for OSCC. Genetic polymorphism frequencies can vary substantially across populations, which may influence the strength of genetic associations with disease. Second, functional studies were not performed to elucidate the molecular mechanisms by which rs11180813 influences NAP1L1 expression or activity. Third, because multiple clinicopathological associations were analyzed across several SNPs, the possibility of false-positive findings resulting from multiple statistical comparisons cannot be completely excluded. Although the present study focused on a limited number of candidate polymorphisms in an exploratory setting, future studies with larger independent cohorts and additional statistical validation will be necessary to confirm the reproducibility of these associations. Future studies incorporating *in vitro* and *in vivo* models will be necessary to clarify the biological significance of this polymorphism.

In conclusion, although NAP1L1 polymorphisms do not appear to significantly influence OSCC susceptibility, the rs11180813 variant is significantly associated with lymph node metastasis and poor tumor differentiation, particularly in buccal OSCC. These findings, together with TCGA expression analysis, suggest that NAP1L1 may be associated with OSCC progression and metastatic behavior.

## Figures and Tables

**Figure 1:**
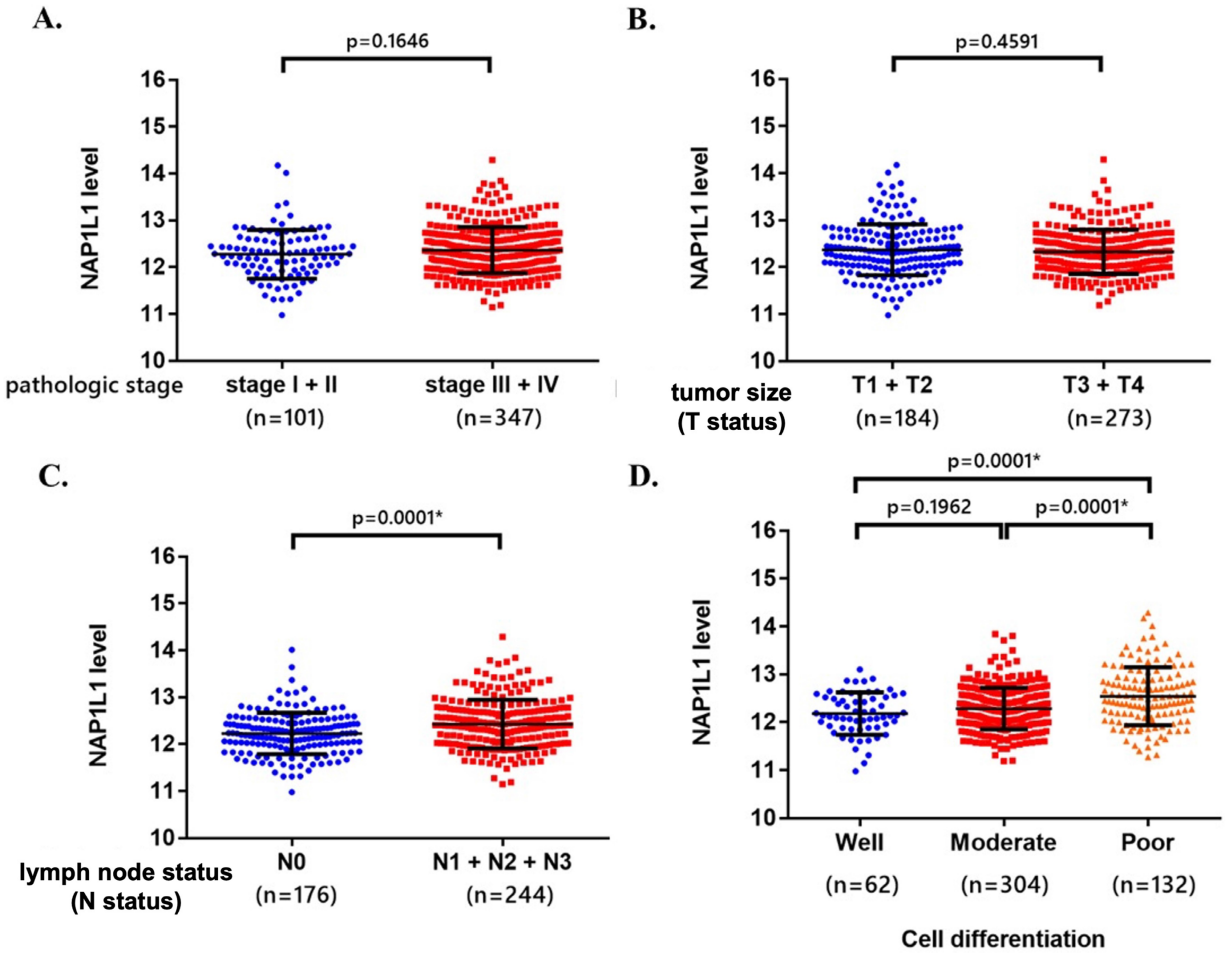
** Association between NAP1L1 mRNA expression and clinicopathological characteristics in the TCGA-HNSCC dataset.** NAP1L1 mRNA expression levels were compared according to (A) clinical stage, (B) tumor size (T status), (C) lymph node status (N status), and (D) tumor differentiation. The data were obtained from The Cancer Genome Atlas (TCGA) head and neck squamous cell carcinoma (HNSCC) dataset using the UCSC Xena platform. Statistical comparisons between groups were performed using the Mann-Whitney U test. *P* values < 0.05 were considered statistically significant. These analyses were exploratory and based on the TCGA-HNSCC cohort.

**Table 1:** The distributions of demographical characteristics and clinical parameters in 672 controls and 910 cases with OSCC.

Variable	Control (N=672)	Patients (N=910)		*p* Value
**Age (yrs.)**	55.87±10.45	56.02±10.16		
>56	336 (50.0%)	488 (53.6%)		0.535
≤56	336 (50.0%)	422 (46.4%)		
**Betel nut chewing**				
No	508 (75.6%)	264 (29.0%)		< 0.001*
Yes	164 (24.4%)	646 (71.0%)		
**Cigarette smoking**				
No	468 (69.6%)	159 (17.5%)		< 0.001*
Yes	204 (30.4%)	751 (82.5%)		
**Alcohol drinking**				
No	402 (59.8%)	542 (59.6%)		0.917
Yes	270 (40.2%)	368 (40.4%)		
**Stage**				
I + II		412 (45.3%)		
III + IV		498 (54.7%)		
**Tumor T status**				
T1 + T2		520 (57.1%)		
T3 + T4		390 (42.9%)		
**Lymph node status**				
N0		646 (71.0%)		
N1 + N2 + N3		264 (29.0%)		
**Metastasis**				
M0		887 (97.5%)		
M1		23 (2.5%)		
**Cell differentiation**				
Well differentiated		135 (14.8%)		
Moderately or poorly differentiated		775 (85.2%)		

N: number. * *p* value < 0.05 as statistically significant.

**Table 2:** The distribution of genotype frequencies in NAP1L1 SNPs in cases of control and OSCC group.

Variable	Control (N=672)	Patients (N=910)	OR ^a^ (95% CI)	*p* Value	AOR ^b^ (95% CI)	*p* Value
**rs11180813**						
GG	592 (88.1%)	792 (87.0%)	1.000 (reference)		1.000	
GC	77 (11.5%)	112 (12.3%)	1.087 (0.798-1.481)	0.596	1.175 (0.795-1.739)	0.419
CC	3 (0.4%)	6 (0.7%)	1.495 (0.372-6.002)	0.571	1.721 (0.307-9.655)	0.537
GC + CC	80 (11.9%)	118 (13.0%)	1.103 (0.814-1.493)	0.528	1.196 (0.815-1.755)	0.360
**rs75354731**						
GG	563 (83.8%)	737 (81.0%)	1.000 (reference)		1.000	
GA	104 (15.5%)	161 (17.7%)	1.183 (0.903-1.549)	0.223	1.214 (0.869-1.695)	0.255
AA	5 (0.7%)	12 (1.3%)	1.833 (0.642-5.234)	0.257	0.683 (0.184-2.531)	0.568
GA + AA	109 (16.2%)	173 (19.0%)	1.212 (0.931-1.578)	0.152	1.181 (0.851-1.637)	0.320
**rs76535500**						
TT	562 (83.6%)	754 (82.9%)	1.000 (reference)		1.000	
TG	106 (15.8%)	150 (16.5%)	1.055 (0.804-1.384)	0.701	0.943 (0.674-1.321)	0.734
GG	4 (0.6%)	6 (0.7%)	1.118 (0.314-3.981)	0.863	1.489 (0.294-7.542)	0.630
TG + GG	110 (16.4%)	156 (17.1%)	1.057 (0.809-1.381)	0.684	0.959 (0.688-1.336)	0.802

N: number. ^a^ The odds ratio (OR) with their 95% confidence intervals were estimated by logistic regression models. ^b^ The adjusted odds ratio (AOR) with their 95% confidence intervals were estimated by multiple logistic regression models after controlling for betel nut chewing, alcohol and tobacco consumption.

**Table 3:** Clinical statuses and NAP1L1 rs11180813 genotype frequencies in cases of OSCC group.

Variable	NAP1L1 (rs11180813)					
	GG (%) (N = 792)	GC + CC (%) (N = 118)	OR^a^ (95% CI)	*p* Value	AOR^b^ (95% CI)	*p* Value
**Clinical stage**						
Stage I/II	366 (46.2%)	46 (39.0%)	1.000	0.142	1.000	0.143
Stage III/IV	426 (53.8%)	72 (61.0%)	1.345 (0.906-1.997)		1.344 (0.905-1.998)	
**Tumor size**						
T1 + T2	455 (57.4%)	65 (55.1%)	1.000	0.628	1.000	0.651
T3 + T4	337 (42.6%)	53 (44.9%)	1.101 (0.746-1.625)		1.094 (0.741-1.617)	
**Lymph node metastasis**						
No	572 (72.2%)	74 (62.7%)	1.000	0.035^*^	1.000	0.031^*^
Yes	220 (27.8%)	44 (37.3%)	1.546 (1.032-2.316)		1.565 (1.042-2.350)	
**Distant metastasis**						
No	773 (97.6%)	114 (96.6%)	1.000	0.524	1.000	0.529
Yes	19 (2.4%)	4 (3.4%)	1.428 (0.477-4.271)		1.423 (0.475-4.264)	
**Cell differentiation**						
Well	126 (15.9%)	9 (7.6%)	1.000	0.021^*^	1.000	0.022^*^
Moderate/poor	666 (84.1%)	109 (92.4%)	2.291 (1.131-4.642)		2.284 (1.126-4.635)	

N: number. ^a^ The odds ratio (OR) with their 95% confidence intervals were estimated by logistic regression models. ^b^ The adjusted odds ratio (AOR) with their 95% confidence intervals were estimated by multiple logistic regression models after controlling for betel nut chewing, alcohol and tobacco consumption. * *p* value < 0.05 as statistically significant.

**Table 4:** Clinical characteristics and NAP1L1 rs11180813 genotype distribution in buccal OSCC patients.

Variable	NAP1L1 (rs11180813)					
	GG (%) (N = 295)	GC + CC (%) (N = 44)	OR^a^ (95% CI)	*p* Value	AOR^b^ (95% CI)	*p* Value
**Clinical stage**						
Stage I/II	147 (49.8%)	16 (36.4%)	1.000	0.098	1.000	0.103
Stage III/IV	148 (50.2%)	28 (63.6%)	1.738 (0.903-3.347)		1.730 (0.895-3.344)	
**Tumor size**						
T1 + T2	177 (60.0%)	24 (54.5%)	1.000	0.493	1.000	0.514
T3 + T4	118 (40.0%)	20 (45.5%)	1.250 (0.661-2.365)		1.239 (0.651-2.356)	
**Lymph node metastasis**						
No	228 (77.3%)	26 (59.1%)	1.000	0.011^*^	1.000	0.011^*^
Yes	67 (22.7%)	18 (40.9%)	2.356 (1.218-4.557)		2.349 (1.213-4.549)	
**Distant metastasis**						
No	291 (98.6%)	43 (97.7%)	1.000	0.642	1.000	0.655
Yes	4 (1.4%)	1 (2.3%)	1.692 (0.185-15.49)		1.661 (0.180-15.35)	
**Cell differentiation**						
Well	57 (19.3%)	4 (9.1%)	1.000	0.109	1.000	0.112
Moderate/poor	238 (80.7%)	40 (90.9%)	2.395 (0.823-6.966)		2.394 (0.816-7.023)	

N: number. ^a^ The odds ratio (OR) with their 95% confidence intervals were estimated by logistic regression models. ^b^ The adjusted odds ratio (AOR) with their 95% confidence intervals were estimated by multiple logistic regression models after controlling for betel nut chewing, alcohol and tobacco consumption. * *p* value < 0.05 as statistically significant.

## Data Availability

The data used to support this study's findings are available from the corresponding author upon request.
